# Dataset for vaginal human papillomavirus infection among adolescent and early adult girls in Jos, Nigeria

**DOI:** 10.1186/s13104-023-06560-3

**Published:** 2023-10-14

**Authors:** Nanma T. Cosmas, Lohya Nimzing, Daniel Z. Egah, Oluranti A. Famooto, Sally N. Adebamowo, Clement A. Adebamowo

**Affiliations:** 1 Department of Medical Microbiology, College of Health Sciences, University of Jos, Jos, Nigeria; 2 Department of Medical Laboratory Science, College of Health Sciences, University of Jos, Jos, Nigeria; 3 Department of Medical Microbiology, Jos University Teaching Hospital, Jos, Nigeria; 4 Department of Epidemiology and Public Health, University of Maryland School of Medicine, 660 West Redmond Street, Baltimore, MD 21201 USA; 5 The Greenebaum Comprehensive Cancer Center, University of Maryland School of Medicine, Baltimore, MD USA

**Keywords:** HPV Infection, Sexual behaviours, HPV vaccine knowledge, HPV types, Adolescent and early adult girls

## Abstract

**Objectives:**

To assess risk factors for HPV infection, determine knowledge about HPV vaccines, assess willingness to receive the HPV vaccine among adolescent and early adult girls in Nigeria, we administered a structured questionnaire. We also collected samples to determine the prevalence and patterns of HPV infections.

**Data description:**

The dataset contains the responses of 205 participants from 10 randomly selected public and private secondary schools in Jos, Nigeria. The data includes information on risk factors for HPV infections such as sexual behaviours, knowledge about HPV vaccine and willingness to receive the vaccine. This is valuable information that can be compared to data from studies in other environments or to determine changes in the pattern of risk factors and HPV prevalence in this population over time.

## Background

Persistent high risk Human papillomavirus (HPV) infection is a necessary cause of cervical cancer [1]. HPV infections are very common and usually transient. The first episode typically occurs shortly after sexual debut in adolescence and early adulthood [2, 3]. Several factors contribute to the risk of HPV infections. These include risky sexual behaviours, sexual hygiene, and substance abuse [4–6]. The World Health Organization recommends immunization of females aged 9–14 years before they begin sexual activity to prevent HPV infection and associated diseases [7]. The widespread use of HPV vaccine is a necessary component in the global efforts to prevent HPV-related diseases. Several factors have contributed to the varying access to HPV vaccine in low- and middle- income countries [8]. In Africa, 31% of nations have included HPV vaccination in their national cancer control strategy [9]. Despite their availability, these vaccines are not yet widely or easily accessible in Nigeria. As a result, there is a need to determine the current prevalence, pattern, and risk factors for HPV infection among young girls in this population.

### Method

We collected data from 205 adolescent and early adult girls. The girls were recruited through a pre-determined aged range of 9 to 20 years old, from 10 randomly selected schools. Details of the enrolment process in the study has been published elsewhere [10]. After enrolment and obtaining informed consent and assent, research nurses from the Jos University Teaching Hospital (Nigeria) interviewed each participant in private using a structured questionnaire. Young nurses were used to encourage the girls to relax and be more opened to participating in this study, particularly in discussions related to sexual matters. The study questionnaire included questions on socio-demographic and family characteristics, sexual preferences, sexual debut, condom use, sexual behaviour including masturbation, menstrual, and personal hygiene practices. To assess the sexual risk factors for HPV infections, we collected data on the sexual history of the girls and information on their sexual partners.

We also collected data on knowledge related to HPV infections and HPV vaccine, and willingness to receive the vaccine. Instead of socio-economic status, we used principal component analysis (PCA) to compute wealth index using data on ownership of household items and availability of household facilities such as type of house they live in, type of toilet facilities, main source of cooking fuel etc. [11]. The questionnaire design was guided by the research questions and literature review [12–14]. We pretested the questionnaire in 10 people to check for correctness and ease of administration. The participants were trained to self-collect vaginal samples which were tested for HPV infection using DEIA/LIPA_25_.

This dataset will contribute immensely to comparisons of HPV infection among other population in Nigeria and other countries. Researchers involved in data mining and analysis of secondary data will also find the dataset useful.

## Data description

The dataset **(data file 1 on** Table 1**)** [15] contains responses to 205 questionnaires from adolescent and early adult girls. Because some of the girls were minors and unable to give consent in their own cognizance, we obtained consent and assent as required [10]. The girls were enrolled from 10 randomly selected schools in Jos, Nigeria after meeting the inclusion criteria of being within the age range and haven provided informed consent and/or ascent. Some 50.7% (104/205) of the participants were recruited from public schools while 49.3% (101/205) were from private schools. The mean age (SD) of the girls was 14.7 (1.97) years with a minimum of 9 and maximum age of 20 years old. Most of the girls (145/205, 70.7%) had attained menarche and the earliest age at menarche was 9 years [11].

**Table 1:** Overview of data set

Label	Name of data file/data set	File types (file extension)	Data repository and identifier (DOI or accession number)
Data file 1	HPV among adolescent and young adult grils_dataset.xlsx	Excel file (.xlsx)	Figshare (10.6084/m9.figshare.20334534.v1) [15]

Only 9.3% (19/186) of the girls reported history of penetrative vaginal sexual intercourse. Most of the girls (57.9%, 11/19) who reported sexual intercourse first experienced it at 15 years of age or less and 15.8% (3/19) experienced it before the age of 10 years. Vaginal douching practices were reported by 16.6% of the girls and most of the girls who practice douching gave ‘It is part of taking a bath’ as the main reason why they engaged in the practice. Most of the girls (89.0%) took their bath at least twice daily when menstruating and 61.4% regularly bathe twice daily. Participants also shared personal clothes (24.9%) and pants (5.4%). Some girls reported history of masturbation, use of toys for masturbation, use of condoms and knowledge about ways to prevent pregnancy.

Of the 205 participants, only 5 (2.4%) had heard about HPV vaccine and only 2 (1.0%) had received the vaccine. Some 32.4% (66/205) girls had no interest in receiving HPV vaccination and the reason for their unwillingness was cost of the vaccine. Almost all the girls (196, 95.6%) would receive the vaccine if it is free or subsidized.

The prevalence of any HPV infections among the participants was 13.2% (27/205) and 15 different HPV types were detected with HPV type 52 being the commonest. Participants had single or multiple HPV infections, with 5 HPV types being the highest number of types of HPV found in multiple infections.

### Limitation

Most participants reported that they had never had any sexual contact. Discomfort about reporting sexual history among participants in this study of young and early adult girls may cause information bias [16]. Our small sample size is small and may impact the assessment of risk factors of HPV infection and characterization of the pattern of HPV types. Participants were enrolled from private and public schools so the results may be different if unschooled adolescent and early adult girls are included in the study.

### Contribution and recommendations for future study

The findings of this study have provided timely evidence-based information that policy makers can harness in the development of effective guidelines for HPV prevention, not only among adolescent and young adults, but targeting the whole population. Future research should target a varied group of teenagers and young adults for prevalence surveys and educational interventions to encourage safe sexual behaviours that reduce HPV infection. For a greater reach, such studies should include both in-school and out-of-school participants, as well as involve important stakeholders such as parents, youth groups, teachers, community leaders, and social media campaigns.

## Data Availability

The data described in this Data note can be freely and openly accessed on 10.6084/m9.figshare.20334534.v1 [15].

## References

[1] Walboomers JMM, Jacobs Mv, Manos MM, Bosch FX, Kummer JA, Shah Kv (1999). Human papillomavirus is a necessary cause of invasive Cervical cancer worldwide. J Pathol.

[2] Winer RL, Lee SK, Hughes JP, Adam DE, Kiviat NB, Koutsky LA (2003). Genital human papillomavirus Infection: incidence and risk factors in a cohort of female university students. Am J Epidemiol.

[3] Castellsagué X, Paavonen J, Jaisamrarn U, Wheeler CM, Skinner R, Lehtinen M et al. Risk of first cervical HPV infection and pre-cancerous lesions after onset of sexual activity: analysis of women in the control arm of the randomized, controlled PATRICIA trial [Internet]. 2014. Available from: http://www.biomedcentral.com/1471-2334/14/551. 10.1186/s12879-014-0551-y PMC4251672 25927224

[4] Panatto D, Amicizia D, Trucchi C, Casabona F, Lai PL, Bonanni P et al. Sexual behaviour and risk factors for the acquisition of human papillomavirus infections in young people in Italy: suggestions for future vaccination policies. BMC Public Health [Internet]. 2012 [cited 2022 Apr 15];12(1):623. Available from: 10.1186/1471-2458-12-623 10.1186/1471-2458-12-623 PMC3490840 22871132

[5] Itarat Y, Kietpeerakool C, Jampathong N, Chumworathayi B, Kleebkaow P, Aue-Aungkul A (2019). Sexual behavior and Infection with cervical human papillomavirus types 16 and 18. Int J Womens Health.

[6] Smith EM, Rubenstein LM, Haugen TH, Hamsikova E, Turek LP. Tobacco and alcohol use increases the risk of both HPV-associated and HPV-independent head and neck cancers. Cancer Causes Control [Internet]. 2010 Sep [cited 2022 Apr 15];21(9):1369–78. Available from: https://pubmed.ncbi.nlm.nih.gov/20401530/. 10.1007/s10552-010-9564-z 20401530

[7] World Health Organization. WHO guidance note: comprehensive Cervical cancer prevention and control: a healthier future for girls and women. WHO Guidelines Note [Internet]. 2013;1–12. Available from: www.who.int.

[8] Ebrahimi N, Yousefi Z, Khosravi G, Malayeri FE, Golabi M, Askarzadeh M, et al. Human papillomavirus vaccination in low- and middle-income countries: progression, barriers, and future prospective. Volume 14. Frontiers in Immunology; 2023. 10.3389/fimmu.2023.1150238 PMC10227716 37261366

[9] Bruni L, Saura-Lázaro A, Montoliu A, Brotons M, Alemany L, Diallo MS et al. HPV vaccination introduction worldwide and WHO and UNICEF estimates of national HPV immunization coverage 2010–2019. Prev Med (Baltim). 2021;144. 10.1016/j.ypmed.2020.106399 33388322

[10] Cosmas NT, Nimzing L, Egah D, Famooto A, Adebamowo SN, Adebamowo CA. Prevalence of vaginal HPV infection among adolescent and early adult girls in Jos, North-Central Nigeria. BMC Infect Dis [Internet]. 2022 Dec 5 [cited 2022 Apr 15];22(1):340. Available from: https://pubmed.ncbi.nlm.nih.gov/35382756/. 10.1186/s12879-022-07215-7 PMC8985253 35382756

[11] Filmer D, Pritchett LH (2001). Estimating Wealth effects without Expenditure Data - or tears. Demography.

[12] Cleland J. Illustrative questionnaire for interview - surveys with young people. Hum Reprod. 2001;3–56.

[13] Mwaka AD, Orach CG, Were EM, Lyratzopoulos G, Wabinga H, Roland M (2016). Awareness of Cervical cancer risk factors and symptoms: cross-sectional community survey in post-conflict northern Uganda. Health Expect.

[14] Ragin CC, Edwards RP, Jones J, Thurman NE, Hagan KL, Jones EA et al. Knowledge about human papillomavirus and the HPV vaccine - A survey of the general population. In: Infectious Agents and Cancer. 2009. p. S10. 10.1186/1750-9378-4-S1-S10 PMC2638455 19208201

[15] Cosmas N, Nimzing L, Egah ZD, Ayo F, Sally NA, Clement AA. Vaginal Human Papillomavirus infection among adolescent and early adult girls in Jos, Nigeria: Dataset [Internet]. 2022 [cited 2022 Jul 21]. Available from: https://figshare.com/articles/dataset/Vaginal_Human_Papillomavirus_infection_among_adolescent_and_early_adult_girls_in_Jos_Nigeria_Dataset/20334534.

[16] Schwartz S, Campbell UB, Gatto NM, Gordon K. Toward a clarification of the taxonomy of “bias” in epidemiology textbooks. Vol. 26, Epidemiology. 2015. 10.1097/EDE.0000000000000224 25536455

